# A Simple Method for Creating Medical Illustrations Using Tablets

**DOI:** 10.7759/cureus.40841

**Published:** 2023-06-23

**Authors:** Masashi Kimura, Yutaro Kondo, Mizuki Hyodo, Tatsuya Kataoka, Kengo Hashimoto

**Affiliations:** 1 Department of Oral and Maxillofacial Surgery, Ogaki Municipal Hospital, Ogaki, JPN; 2 Department of Oral and Maxillofacial Surgery, Toyokawa City Hospital, Toyokawa, JPN; 3 Department of Maxillofacial Surgery, School of Dentistry, Aichi Gakuin University, Nagoya, JPN

**Keywords:** intraoperative photograph, digital, surgical record, illustration, computer assist

## Abstract

Medical illustrations are defined as illustrations that contain and convey medical information. Illustrations in surgical records play a pivotal role not only in recording medical information but also in sharing surgical information, improving own surgical skills, and teaching young doctors. However, we believe that creating a medical illustration from a blank sheet of paper is challenging for beginners. The computer-assisted illustration technique proposed in this article not only saves time but also provides accurate and easy-to-understand medical illustrations. This technical note aims to introduce a simple and easy method for creating medical illustrations by tracing intraoperative photographs using an iPad™ and an Apple Pencil™. We believe that “anyone can draw” detailed, easy-to-understand medical illustrations using the present method, and we hope that many young doctors will actively create medical illustrations.

## Introduction

Medical illustrations are defined as illustrations that contain and convey medical information and are commonly used in surgical records, figures of medical journals, and instructions for patients to increase the readability and comprehensibility of these documents. Opportunities to draw medical illustrations by conventional methods using pens and brushes have been decreasing owing to the widespread availability of electronic medical records. However, illustrations in surgical records are crucial not only in recording medical information but also in sharing surgical information, improving one’s surgical skills, and teaching young doctors.

Recently, with the development of digital equipment, including iPad™ and Apple Pencil™, we believe it is now easier to create medical illustrations using these devices. This article introduces a simple and quick method for creating medical illustrations by tracing intraoperative photographs using an iPad™ and Apple Pencil™ to save time and effort. This technical note aims to guide oral and maxillofacial surgeons who want to create their own digital medical illustrations, even those with a self-proclaimed lack of artistic ability.

## Technical report

Electronic devices and software

The use of a stylus pen with a tablet provides ease of drawing and a more natural feel than mouse clicking on a computer [1]. Thus, we recommend the use of these electronic devices. One of the major tablets and stylus pen are iPad™ with Apple Pencil™ (Apple, Cupertino, CA, USA). Another option is a detachable keyboard or a tablet computer, such as Microsoft Surface Pro with a digital pressure-sensitive pen called Surface Pen (Microsoft Corp., Redmond, WA, USA) [2].

Although many illustration software programs are available, such as Procreate (Savage Interactive Pty Ltd., Hobart, Tasmania, Australia), Adobe Photoshop (Adobe Systems Inc., San Jose, CA, USA), and Adobe Illustrator (Adobe Systems Inc., San Jose, CA, USA), we recommend software that can use layers. In addition, software that allows the use of pressure-sensitive pens and blending brush tools is recommended.

We created the medical illustrations in this technical note using an iPad™ with Apple Pencil™ and Procreate software [3].

Steps to create medical illustrations using iPads™

Scanning an Intraoperative Photograph

**Figure 1:**
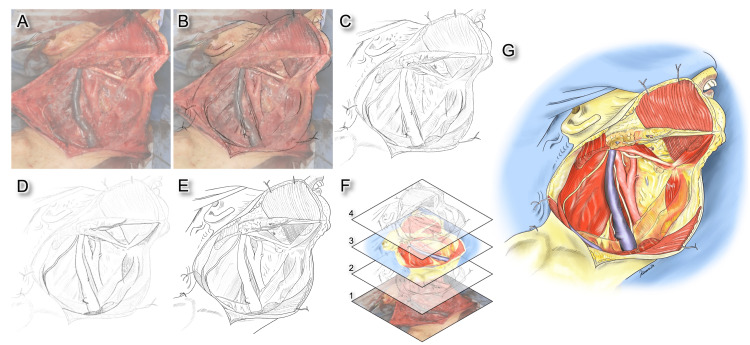
Each step to create the medical illustration from an intraoperative image. (A) Import an intraoperative image into an iPad™ and adjust the opacity. (B) Create a new layer above the image and trace the imported image with a pencil-like brush. (C) Created a basic sketch. (D) Lower the opacity of the drawn line and create a new layer above this rough draft layer. On this new layer, draw the final line with the pen brush. (E) Created line art. (F) Structure of layers. Layers in Procreate software act like sheets of tracing paper and make it possible to stack image elements on top of each other (1, intraoperative image; 2, draft drawn by a pencil-like brush; 3, coloring layer; and 4, line art). (G) The final version of the illustration after coloring.

For beginners, it is challenging to create medical illustrations on a blank sheet of paper. Therefore, the authors import an intraoperative photograph into an iPad™ and use it to create medical illustrations. Adjust the image opacity once the intraoperative photograph has been imported (Figure 1A). Following this step, create a new layer above this image and trace the imported image with a pencil-like brush (Figures 1B, 1C). This technique allows the creation of a basic sketch with an accurate scale. After tracing the operative image, lower the opacity of the drawn line and create a new layer above this rough draft layer. On this new layer, draw the final line with the pen brush (Figures 1D, 1E). This step allows for the removal of unwanted strokes. Furthermore, additional information, including anatomical structures that are hidden in the clinical images and surgical instruments and techniques, should be added to this step. We believe that the line art created by these procedures is of sufficient quality to be used as illustrations for surgical records at this step. To create more detailed and comprehensible illustrations, coloring is done in the next step. The structure of the layers and the final version of the illustration after coloring are shown in Figure 1F and Figure 1G, respectively. The process of creating the present medical illustration is shown in Video 1.

**Video 1:** The process of creating the medical illustration.

Coloring to Create a Three-Dimensional Effect

We believe that the keys to creating three-dimensional illustrations are the strength of the lines and the shading of the colors. A pressure-sensitive pen is useful for creating lines with varying weights (thickness). In the case of creating uniform lines, the illustration tends to become monotonous, making it difficult to express a sense of three-dimensionality. Therefore, we made the lines in the front thicker and the lines in the back thinner using a pressure-sensitive pen.

**Figure 2:**
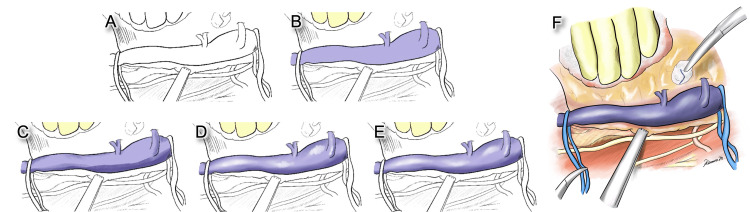
Each step of the coloring procedure for expressing three-dimensionality. (A) Created line art. (B) Painting the base color with a color between the lightest and darkest parts of the structure. (C) Painting the darkest area of the structure in a dark color. (D) Adding the highlights in white. (E) Blending in the highlight and shadow with a blending brush. (F) The final version of the illustration after coloring.

The coloring procedure for expressing three-dimensionality is as follows: The first step is to paint the base color with a color between the lightest and darkest parts of the structure (Figures 2A, 2B). The second step is to paint the darkest area of the structure with a shadow in a color that is even darker than the base color (Figure 2C). The third step is to add the highlights on the lightest area in white (Figure 2D). The final step is to blend in the highlight and shadow with a blending brush (Figure 2E). Our method uses only three colors for a single structure: base, highlights, and shadows. Figure 2F shows the final version of the illustration after coloring, and Video 2 shows the process of the present medical illustration.

**Video 2:** The process of coloring to create a three-dimensional effect.

## Discussion

Treatment strategies for head and neck tumors and orthognathic surgery have evolved recently, including preoperative computer-assisted surgical planning [4-6]. While the importance of preoperative treatment planning has been widely recognized, a detailed description of intraoperative findings using easy-to-understand illustrations also has pivotal roles and may contribute to improving treatment quality. Furthermore, the role of medical illustration in surgical records is to record surgical information, improve one’s surgical skills, and educate young doctors [1,2]. However, we believe it is challenging for beginners to create medical illustrations from a blank sheet of paper. The computer-assisted illustration technique not only saves time but also provides accurate and easy-to-understand medical illustrations [2]. The previous reports include methods of converting radiologic images to line art using photo editing software and digitization of hand drawings [1,2]. The method proposed by the authors differs from previously reported illustrations in that it uses intraoperative images to create a basic sketch with an accurate scale.

We believe that the most important advantage of using computer-assisted illustrations is that it is easy to modify the illustrations repeatedly, implying that the present technique is quicker than the traditional method for creating high-quality results. Another advantage compared to intraoperative images is that surgeons may add the hidden anatomical structures of intraoperative images to the illustration. In addition, the instruments used during the surgery can be added to the illustration. This additional information might be useful for teaching young surgeons. Therefore, the most significant aspect of creating illustrations for surgical records is not to create the photographic illustration but to add additional information that the clinical images could not contain. To prepare figures for publications, it is also easy to determine the required final resolution and the direct digital output format [2]. In addition, these data can be stored permanently. Thus, computer-assisted illustrations have many advantages. However, there are some limitations to the present method. One disadvantage is the initial cost. The introduction of digital equipment, such as iPad™ with Apple Pencil™, is expensive and can be burdensome for young physicians. However, it would be lower than requesting a professional illustrator to do the work. Second, it takes time to learn to operate the software. However, procreate software is a very easy-to-use drawing application. Thus, it generally takes about a few days to become familiar with the software in our experience. Third, intraoperative photographs are essential for this technique. However, with the recent advancement of operating room facilities, an increasing number of hospitals have the capability of recording videos. Thus, not only intraoperative images but also images created from intraoperative videos can be used for this technique. We hope that many surgeons will use the present method and that it will lead to further advancement.

## Conclusions

Creating many detailed surgical records at a young age is highly beneficial for improving one’s surgical technique and may contribute to improving overall treatment quality. We believe that “anyone can draw” detailed, easy-to-understand medical illustrations using the present method, and we hope that many young doctors will actively create medical illustrations.
